# Shaping healthy habits in children with neurodevelopmental and mental health disorders: parent perceptions of barriers, facilitators and promising strategies

**DOI:** 10.1186/s12966-019-0813-6

**Published:** 2019-06-26

**Authors:** April Bowling, Rachel E. Blaine, Raghbir Kaur, Kirsten K. Davison

**Affiliations:** 1 Department of Public Health and Nutrition, Merrimack College School of Health Sciences, 315 Turnpike Street, Office 401, O’Reilly Hall, North Andover, MA 01845 USA; 2 California State University, Long Beach, USA; 3 Harvard T.H. Chan School of Public Health, Boston, USA

**Keywords:** Youth, Parenting, Autism, Mental illness, Physical activity, Diet, Sleep, Screens

## Abstract

**Objective:**

Prevalence of pediatric neurodevelopmental and mental health disorders (ND/MHD) is increasing in the United States and globally. ND/MHD are associated with higher risk of poor dietary, physical activity (PA), screen, and sleep habits in youth, contributing to elevated lifetime chronic disease risk. ND/MHD symptoms can present unique challenges to parenting, create competing parenting priorities, and may decrease parental capacity to instill healthy habits. Unfortunately, literature characterizing parenting of health habits in youth with ND/MHD is sparse. The objective of this study was to describe barriers to, facilitators of, and practical strategies for parenting healthy lifestyle habits in children and teens with ND/MHD.

**Methods:**

We conducted semi-structured interviews with parents whose children with diagnosed ND/MHD were attending a Boston-area therapeutic day school serving K-10th grade. Interviews allowed parents to focus on parenting PA, diet, sleep, and/or screen habits as context for questions. Interviews were transcribed, double-coded using constant comparative methods, and summarized into themes using NVivo 11.

**Results:**

We interviewed 24 parents; average age of their child with ND/MHD was 11.2 years (range: 8–15). Most had a son (75%) with multiple ND/MHD (88%); diagnoses included autism spectrum disorder (50%), attention deficit-hyperactivity disorder (67%), anxiety (67%), and other mood disorders (58%). Major barriers to parenting all types of health habits included depleted parent resources, child dysregulation, lack of supportive programming available to children with ND/MHD, and medication side effects. Major facilitators included participation in specialized therapeutic options, adaptive community programs and schools, as well as parents’ social capital. Effective parenting strategies included setting clear, often structural boundaries, using positive reinforcement, allowing agency by presenting healthy choices, and use of role modeling to promote healthy habits. Almost one third of parents extensively discussed the role of pets or therapy animals as key to establishing and maintaining healthy routines, particularly PA and screen-time management.

**Conclusions:**

Parenting healthy habits in children with ND/MHD is difficult and is undermined by competing demands on parenting resources. To reduce chronic disease disparities and promote health in this population, future research must better adapt existing health promotion materials and programs to more practically support parents in multiple settings including home, schools and community organizations.

**Electronic supplementary material:**

The online version of this article (10.1186/s12966-019-0813-6) contains supplementary material, which is available to authorized users.

## Introduction

Pediatric neurodevelopmental and mental health disorders (ND/MHD) occur in over a quarter of those under the age of 18 in the United States, and prevalence is growing globally [1]. Neurodevelopmental diagnoses, including autism spectrum disorders (ASD) and attention-deficit/hyperactivity disorder (ADHD), affect approximately 15% of U.S. youth [2]. Meanwhile, other mental health disorders span diagnoses such as anxiety, mood, and schizoaffective disorders, with about 22% of U.S. children and adolescents (hereafter children) suffering severe impairments from their diagnosed condition [3]. Importantly, estimates of ND/MHD comorbidity are very high; over 40% of children with ASD are also diagnosed with ADHD or anxiety, while approximately 25% of children with ADHD have anxiety, and 50% have additional learning disorders [4].

Health disparities faced by children with ND/MHD are considerable. Several studies have found that children with diagnoses such as ASD, ADHD, bipolar, depression, and anxiety display high levels of unhealthy lifestyle habits, such as low physical activity levels [5], poor diet [6], disrupted sleep [7, 8], and elevated screen time [9]. These unhealthy habits in childhood likely contribute to elevated chronic disease risk [10]. For example, it has been found that risk of obesity is twice as high among children with ASD relative to their neurotypical peers, and prevalence in the population with ASD increased with age while it decreased for the reference group [11]. Meanwhile, adolescents with ADHD were found to have approximately four times the incidence of type 2 diabetes over a 3 year period, compared to those without ADHD [12]. Similar patterns are observed among children with ND/MHD for a variety of cardiovascular disease risk factors, including hypertension and metabolic syndrome [12].

Research has begun to elucidate how parents shape children’s health habits that predict future chronic disease risk, particularly dietary intake, screen time, physical activity, and sleep hygiene (the practices and habits enabling good nighttime sleep quality). Studies have also described the differences in parenting behaviors among families with typically developing children, those with ND, and those with MHD, particularly externalizing behavioral disorders. For example, Garner et al. utilized data on over 14 thousand Canadian children and parents to examine differences in parental consistency, and found that parents of children with comorbid ND/MHD were less consistent than parents of children with ND only or no disability [13]. They were also less positive and less effective than parents of children with no disorders [13]. One important takeaway of this study is that the additional burden of parenting difficult externalizing behaviors – so common among children with ND/MHD - may prove especially challenging to parents’ ability to apply the positive and consistent parenting methods that are especially crucial to instilling healthy habits.

Unfortunately, very few studies have examined parenting of health habits in children with ND/MHD. Despite this, or perhaps partially because of it, as a plethora of misleading information and strategies that are not evidence-based have emerged, particularly around use of diet to control symptoms, and are widely available through a variety of social media and internet sources. While a small number of studies have examined health parenting of children with ASD or ADHD, almost all of these studies have focused on children with a single diagnosis (i.e., ASD or ADHD in the absence of any other mental health disorder). However, with comorbidity estimates for ND/MHD over 50% in most studies and diverse symptom constellations associated with multiple diagnoses complicating parenting approaches, this represents a major gap that may limit the effective design of interventions and support aimed at improving lifestyle habits and health outcomes for children with ND/MHD and their families.

Qualitative research methods are appropriate to explore and gain an understanding of the realities involved in parenting health habits of children with ND/MHD. Understanding the realities involved in parenting health habits of children with ND/MHD, including those with multiple diagnoses, will aid the development of more effective strategies to promote healthy habits and improve clinical, community and school supports. Therefore, the aims of this qualitative investigation were to 1) identify overarching barriers and facilitators to parenting healthy habits reported by parents of children with ND/MHD and associated behavioral challenges, and 2) identify strategies these parents are currently employing to address barriers and create and maintain healthy habits in their children.

## Methods

### Design

The qualitative design of this study involved a brief screening survey, followed by individual interviews conducted by trained research staff. Written consent was obtained for all participants, and all study protocols were approved by the Harvard T.H. Chan School of Public Health Institutional Review Board.

### Participants

Participants were parents who had at least one school-age child with one or more neurodevelopmental and mental health diagnosis, and were recruited through a therapeutic day school affiliated with Boston Children’s Hospital in Massachusetts. The school serves children who have been diagnosed with ND/MHD with externalizing behaviors that prevent their success in public school special education programming, but do not have commensurate intellectual disabilities. Our recruitment strategy ensured that participants were parents of children with severe impairments who were mostly living at home and taking part in regular parenting interactions around health habits. The recruitment school serves children in kindergarten through 10th grade, increasing the developmental representation of the sample. There were just over 100 eligible participants in the sampling frame, since only one parent per family was allowed to participate.

Recruitment occurred during the 2016–2017 school year. Information was distributed at parent support groups, through email, and via hard copy letters sent home by the school to families of all students. Interested parents completed a brief online demographic survey and consent form, after which they were contacted by research staff to schedule an interview. Recruitment continued until there was no online consent completed by additional potential participants for 2 weeks, representing exhaustion of the sampling frame. Participants received a $15 gift card after interview completion.

### Data collection

Four trained research staff conducted the interviews using a semi-structured interview guide, including prompts, developed from an initial, unpublished literature review of studies examining parenting children with disabilities, as well as two pilot interviews. Parents were instructed to only answer questions in relation to their child attending the therapeutic day school, even if they had multiple children with ND/MHD. After initial questions regarding lifestyle parenting in general, parents were asked to choose two lifestyle parenting topics from a choice of four (encouraging the child’s healthy eating; getting enough exercise; limiting screen time and media use and; getting a good night’s sleep) to frame the interview. The interviews were conducted in person, by phone, or through video-conference to reduce barriers to participation. They lasted from 40 to 75 min, and were audio recorded. The interview guide is included as supplementary material (Additional file 1).

### Data analysis

Interviews were transcribed verbatim, de-identified and cleaned, and analyzed in NVivo 11.0. We analyzed data thematically using modified grounded theory methods [14]. Initial main theme codes developed a priori based on existing literature and research aims included general parent-level barriers and one sub-theme (depleted reserve capacity), general child-level barriers and one sub-theme (dysregulation), facilitators, and strategies. Ten transcripts were selected at random and two researchers (AB, RK) coded each transcript to ensure intercoder reliability. Any disagreements were resolved during joint review meetings with a third researcher (RB). We made subsequent additions and revisions to the codebook as inductive sub-themes emerged during the initial 10 transcript review process (AB, RB, RK). After the codebook was finalized, one reviewer (RB) coded each of the remaining transcripts with a secondary reviewer (AB). We then organized codes thematically with representative quotes extracted; the full research team cross-validated provisional findings (AB, KD, RB, RK).

## Results

### Participant characteristics

A total of 24 parents completed interviews; one parent completed the initial online screening but declined to participate in an interview. Parent and child characteristics are captured in Table 1. The sample identified almost entirely as white mothers; 62% were married or cohabitating at the time of the interview. Of the 15 parents reporting having more than one child, seven had a second child with special needs. The majority of parents (87.5%) reported that the target child had multiple diagnoses.

**Table 1:** Participant and Child Characteristics

Characteristic	Participants (*n* = 24)
Parent	
Married or cohabitating	15 (63%)
Non-Hispanic White	23 (96%)
Mothers	23 (96%)
Completed college or graduate school	18 (75%)
Child qualifies for free or reduced cost-lunch	3 (13%)
More than one child	15 (63%)
More than one child with special needs	7 (29%)
Child	
Age (yrs)	11.6 (2.2)
Male	18 (75%)
Diagnoses	
ASD	12 (50%)
ADHD	16 (67%)
Anxiety	16 (67%)
Mood	14 (58%)
Multiple	21 (88%)

Diet was the most common health behavior chosen to discuss by parents (*n* = 15), followed by physical activity (*n* = 11), screens (*n* = 10) and sleep (*n* = 6). Results were organized around three major themes: 1) parent and child-level barriers, 2) parenting facilitators, and 3) strategies for parenting healthy habits in children with ND/MHD. These themes were then divided into important sub-themes based on number of common sources (Table 2). Quotes include a non-identifiable participant code to allow for source differentiation.

**Table 2:** Themes and Sub-themes

Theme	Sub-themes
Barriers – Child Level Factors	Behavioral and Sensory Dysregulation
	Contextual Limitations
	Medication Side Effects
Barriers – Parent Level Factors	Challenges Modeling and Maintaining Healthy Habits
	Depleted Reserve Capacity of Parents
Facilitator Themes	Knowledge and Use of Specialized Therapeutic Options, Community Programs and Schools
	Intrinsic Family, Parent and Child Attributes
Strategies	Allowing Agency
	Clear and Consistent Boundaries
	Positive Reinforcement
	Pets and therapy animals
	Preparation and Review
	Family Engagement and Positive Role Modeling

### Theme 1. Barriers to parenting healthy habits

Parents reported both child and parent-level barriers to parenting healthy habits in their children with ND/MHD.

#### Behavioral and sensory dysregulation (child-level)

All parents related barriers to instilling healthy habits in their children due to the child’s dysregulation and symptoms related to their diagnoses. Parents reported child behaviors such as aggression, agitation, withdrawal, suicidal ideation, opposition, and increased anxiety as impacting their abilities to influence and set boundaries around their child’s lifestyle habits.

“Taking (the computer) away is the hardest, because he will call people names, he will stomp, he will throw things. He will calm down eventually, but it takes a whole effort to try to take it away from him… People just don't understand that a neurotypical kid's meltdown and a neurodivergent kid's meltdown are completely different planets.” [P14]

“It's always been a big struggle with him to get him in any organized sport activity, because he feels that pressure because his biggest thing is anxiety.” [P25]

“There are times where I say we're going for a hike, and she will start screaming bloody murder and refusing to go. It is complete hell. I have not been able to figure out why sometimes it works, and sometimes it doesn't. I'd say 90 percent of the time is resistance.” [P16]

Another common barrier to adoption of healthy habits identified by parents was children’s resistance to change, rigidity, and/or lack of intrinsic motivation to change, which parents felt was a facet of their child’s diagnoses. Sensory issues, which are often experienced by children with ND/MHD, were also reported by parents to be a barrier to healthy habits ranging from food selection to exercise to sleep.

#### Contextual limitations (child-level)

Most parents cited contextual limitations to parenting healthy habits that were unique to a child with ND/MHD and associated behavioral challenges, such as different peer and school norms around weight-related behaviors. One such commonly cited contextual limitation was the extraordinarily long school commutes to attend special education programs, which increased screen time and decreased physical activity time. Another important contextual limitation reported by several parents was lack of applicable health programming or clinical expertise among lifestyle experts such as registered dietitians, personal trainers, and sports coaches.

“We [did] take him to a nutritionist, which helped zero…I think they spoke with him as if he were just a neurotypical child [not] taking into account that he has disabilities. That might have had something to do with it. It wasn’t helpful for us…She was very nice, but I don't think she really understood.” [P01]

This resulted in frustration when some parents tried to obtain health counseling to improve their child’s health habits. Also, very few parents were able to locate any adaptive sports or physical activity programming such as swimming lessons that could accommodate children with behavioral challenges.

#### Medication side-effects (child-level)

About one-fourth of parents cited the side-effects of their children’s psychiatric medications as negatively impacting their ability to parent health habits, particularly around food.

“Because he was always in the twentieth percentile or below for weight, but then right when his issue started happening…and he started medication…he’s gained 30 pounds in 3 months. Then ever since then with all the other medications, he’s never been able to lose the weight.” [P01]

#### Challenges modeling and maintaining healthy habits (parent-level)

The vast majority of parents identified ND/MHD-specific barriers to modeling, initiating and maintaining healthy habits in their children. For example, parents often articulated that extremely high levels of consistency were necessary to maintain healthy habits without constant battles. This high level of consistency bordered on rigidity, diminishing the pleasure available in their children’s already difficult lives.

“I think this is one reason we don’t push him too hard at home, because he does seem happy and content. I know he gets pushed all the time at school to fit expectations. We don’t want him to be overwhelmed.” [P17]

“Sometimes that’s [online] where he feels the world doesn’t judge him. Sometimes that’s when I disagree with my husband. ‘Just give him half an hour more.’ That’s the only time we disagree, if he’s havin’ a really hard time.” [P14]

Many parents were open regarding their use of unhealthy foods and additional screen time as rewards for socially-appropriate behavior. Most recognized this as a problem, but felt that they lacked practical alternatives.

“We use it as incentive sometimes to get him to do other things. We'll offer him a dessert as an incentive for excellent behavior at school or something like that, and so in doing so, we're giving him the junk that we're actually trying to limit.” [P18]

#### Depleted reserve capacity (parent-level)

The most commonly cited parent-level barriers were depleted emotional, social, physical, and economic reserves, which together form a bank of psychosocial resources known as reserve capacity [15]. In fact, most parents named the inherent exhaustion related to the unique demands of parenting a child with ND/MHD as an immense obstacle to prioritizing parenting of health habits.

“If it was a scale of zero to five, with zero being no stress and five being stressed, I would say [our family stress is] probably an eight…I love him to death, but [my child is] extremely stressful. Every day you're wondering what's he gonna do?” [P27]

“I'd say that's why exercise is the hardest…just the battle of spending the next 45 minutes trying to argue with her and cajole her and convince her to go outside, we either have run out of daylight and time, or I am just too exhausted and do not want to do battle in order to take a walk.” [P16]

“I think the parents of typically developing kids, if they want to go to the gym, they can do that, or if they want to send the kids outside to play with other kids, they don't have to worry about their child making appropriate choices or getting into the kind of situations that we have to worry about with our child.” [P15]

Parents particularly felt that prioritizing healthy habits was most difficult when they believed that other competing needs were more pressing, such as preventing school absenteeism or aggressive behaviors towards peers. Parents felt they needed to pick their battles carefully to get any compliance from their children with ND/MHD, and healthy habits were not the most important battle to fight.

“I just kinda feel like, “You know what? There’s all kinds of typical kids that survive on macaroni and cheese, so I’m not gonna make that like a focal point when she has so many other more pressing needs”.” [P09]

Many reported a sense that they were operating with depleted social resources due to their child’s needs, resulting in family strife and job stress.

“Unfortunately, there haven’t been a lot of supports for us, for my husband and I, specifically, and it’s taken a toll over the years. We started seeing a marriage counselor, and one of the things that she said to us is, ‘The biggest issue you guys have is that you’ve been doing this by yourselves without any support.” [P04]

“I'm gonna get all tearful, but I've had to realize all my PTSD from parenting (my child) because I used to get called twice a week, having to leave my job to go get her because they wouldn't allow her to be at school anymore. Just being able to function on basic day to day things like go to my job and stay there and not have to rush out of meetings and drive to get to the school before the police got there just—that's not a good way to live.” [P16]

Both ND and MHD have genetic and inter-generational physiological causal drivers, which were acknowledged by several parents. A high proportion of parents cited either their own mental health challenges or a second child with special needs as unique drivers of reserve capacity depletion.

“Four out of five of us have anxiety issues…. Right now, the current thing is trying to get [my other child with special needs] an IEP as she goes back into public schools. Just a lot of responding to stressful things. The planning, trying to get her to—trying to get ready for IEP meetings and that whole battle.” [P03]

### Theme 2: facilitators of parenting healthy habits

Parents were able to identify a variety of facilitators that improved their ability to parent healthy habits in their children. Some of these facilitators were inherent to the family, such as intrinsic child characteristics (symptom decline with maturation) or family income, but most were modifiable, such as knowledge of available resources and support and therapeutic options. In keeping with the aims of this study, only modifiable facilitators applicable across lifestyle habits are reported here.

Almost all parents emphasized the need to actively explore and engage specialized therapeutic options, community programs and schools to optimize their children’s potential and diminish parent stress, setting the stage for better health behavior parenting.

“Being at [a therapeutic school] now, there's been no phone calls [from police or the school about my child] since we started. That's such a tremendous gift to know that she's safe…I'd say that since joining the [therapeutic school] community, our stress levels have gone down tremendously.” [P16]

In several cases, parents associated these resources with improvements in their child’s lifestyle habits, including sleep, physical activity and diet.

“I’m really thrilled that she’s at [a therapeutic school] and that they have the bikes and adaptive PE there. She does get that regular physical activity as part of her school day.” [P09]

Parenting programs offered by psychiatric clinicians and schools were commonly reported as a powerful facilitator of parenting healthy habits. While not directed specifically at parenting health habits, these training programs were cited as helping parents identify many of the strategies that they employ day-to-day to manage health habits in their children, including consistency, use of clear and structural boundaries, positive reinforcement and reward systems, and role modeling.

“My husband and I did [parenting training]. That really helped a lot in terms of just keeping us cognizant of how to talk to things. Usually, what I’m thinking is, “How can I make this more of a positive thing?” Like, “It was really great how you got off the computer yesterday. That made it possible for you to do this today.” Or, “It was great how you did your homework first, so you had your full computer time. Let’s keep it up”.” [P03]

### Theme 3: strategies to promote healthy habits in children with behaviorally challenging ND/MHD

#### Allowing agency

Parents were candid about parenting healthy habits by making conscious tradeoffs (i.e. allowing more screen time than optimal in exchange for eating healthy foods at dinner) based on their child’s particular challenges. Parents also emphasized that it was necessary to limit children’s options when allowing them agency in making lifestyle choices, but that their child’s ability to take ownership for their choices was critical to creating long-lasting healthy habits. They also emphasized that taking the time to negotiate and presenting some flexibility was key to gaining child buy-in and avoiding melt-downs and oppositional behaviors.

“I try and involve her in the cooking as much as possible so that she feels some ownership over what's being made and how it's being made and everything.” [P16]

“What I’ve done is I made cards. One of them has a bagel on it, and one of them has cereal on it. The rest are healthier options, like yogurt and eggs and things like that. He gets to pick which days he wants those two, I would say, unpreferred [sic] breakfast. I think the power gives him a little bit more control.” [P23]

“We’ll let her––if she says she’s anxious and she needs a few minutes, we’ll give her a few minutes, but then we tell her she needs to come to the dinner table or she needs to go to sleep.” [P11]

#### Clear and consistent boundaries

Parents made clear that such agency and limited negotiation should occur within clear, often structural limits (e.g. automatic screen-time controls, internet disabling software, not having certain foods in the house at all), which helps minimize oppositional behaviors and embed consistent behavioral routines that promote healthy habits in their children.

“We usually, we tend to close it down, the internet down. We also just make her give us… her phone, and her iPad, and her laptop (*at night*).” [P03]

“For example, just not having the cookies in the house versus saying you can only have one and fighting, I try to keep the conflict away that way, by making it more structural.” [P20]

#### Preparation and review

One helpful strategy that parents reported learning from parent training classes, was planning ahead and previewing situations with their children. This helped to both ensure that they had the tools to make the healthiest choices (in the cafeteria for example) and to avoid potential pitfalls from frustration or unexpected changes (e.g. the discontinuation of a favored healthy menu item). When children made poor choices, parents reported consistently reviewing the consequences of those choices (e.g. upset stomach, inability to sleep, headache, etc.).

#### Family engagement and role modeling

Another key strategy reported by most parents was the use of family activities and positive role modeling to help their children adopt the healthiest habits possible within the context of their challenges.

“We try to do it for [whole] the family [or] he goes, “Oh, well, [my brother’s] not doin’ it.” I said, “Hey, look at all of us. We’re [walking] as a family”.” [P14]

#### Positive reinforcement

Finally, all parents reported the use of positive reinforcement as crucial to the adoption and maintenance of their children’s healthy habits. Structured reward systems were a common approach (e.g. sticker charts), as was verbal praising of healthy choices.

Almost a third of parents interviewed extensively discussed the role of pets or animal therapy in the context of their strategies to promote healthy habits. Although mainly discussed as positive reinforcement for good choices, animals were also discussed as key to maintaining healthy routines (physical activity), performance of healthy activities as a family, and role modeling of healthy choices by parents (stress relief and physical activity).

“We just recently, like I said, got a dog a couple months ago. She’s really brought us together, I think, in a nice way where we all go to the dog park together. We all take her on walks together. When we travel, we go on hikes and stuff. We’ll go to the beach in the summer. That kind of thing. We do more fun things outside like that.” [P02]

“I live in a condo association with four units. My neighbors downstairs will watch her if I need them to…They allow me to use their dog and their child as motivation for her, like as a reward for her if she’s upset. Sometimes in the morning, she and my mom get into it. She’ll refuse to get on the bus for my mom. My mom is texting me at work, being like, “Oh, she’s not getting on the bus.” I’ll text my downstairs neighbor and say, “Can she come pet the dog and say hi to your daughter?” Then that’s enough motivation for my daughter to shape up and behave, that she gets the dog. I have neighbors like that, that are very helpful. They let us walk their dog, which is really good for her.” [P09]

“I would say the primary thing for both me and [child] is we foster kittens right now. We have several of our own cats…We volunteer through a shelter, and then they're adopted out. That tends to be pretty Zen for both of us. There's nothin' like savin' a cute, little, furry life to get you through the day.” [P12]

## Discussion

Parents of children with ND/MHD face significant challenges, particularly when their child has both ND and MHD. These challenges can make prioritizing and effectively parenting healthy habits extremely difficult. At the same time, children with these diagnoses face elevated lifetime risk of a variety of cardiometabolic diseases, making acquisition and maintenance of healthy nutrition, physical activity and sleep habits especially important. In this study, we interviewed parents of children with ND/MHD to identify specific barriers, facilitators and strategies for parenting healthy lifestyle habits in this special population.

The most commonly cited child-level barrier was the child’s behavioral and sensory dysregulation. Parents reported the need to choose their battles carefully, and – while acknowledging concern about weight gain and unhealthy lifestyles – universally expressed that keeping their children safe, in school, and functional took precedence over concerns about health habits. Given this reality, it was unsurprising that all parents interviewed cited various forms of reserve capacity depletion as a key parent-level barrier to more effective parenting of health behaviors in their children. Additionally, most parents discussed the lack of specialized services and accessible community programs as an exacerbating factor, isolating their family and reducing potential support and peer learning around healthy lifestyles.

This is a troubling, but not unexpected finding. Individuals draw upon reserve capacity and social capital – a finite bank of psychosocial resources - in response to stressors [15]. As a finite resource, both can be depleted. Low reserve capacity is theorized to create a negative emotional and physiological cascade that amplifies chronic disease risk from environmental and personal factors [15]. The reserve capacity framework has been extended to include health habits, after being shown to be an independent predictor of early mortality risk in vulnerable populations [16]. It is conceivable that the high demand of raising a child with multiple medical, social, behavioral, and learning challenges increases the risk of accelerated depletion in parents, compromising their ability to devote resources to parenting of healthy habits. Likewise, navigating life with symptomatic challenges could do the same for reserve capacity in children, diminishing their ability to focus inter- and intrapersonal resources on healthy choices.

Despite these obstacles, most parents were able to identify specific resources that acted as facilitators of parenting healthy habits. Parent training programs offered through schools and clinics were cited as especially important, in order to learn strategies specific to children with ND/MHD. Parents also said that the ability of their child to attend a specialized therapeutic school had benefits directly related to healthy habits, since health behaviors could be reinforced by specially trained staff and adaptive programming, alleviating some parental burden. One of the most important facilitators was a strong social support network comprised of at least some individuals familiar with the challenges of raising a child with ND/MHD. The ability to rely on close friends, family, and/or support groups were cited by many parents as critical to enabling effective parenting and reinforcing healthy habits in children outside the home.

Given that several parents had found the generalized advice given by health experts such as registered dietitians unrealistic and unhelpful, it was particularly important to elucidate successful strategies that parents utilize to instill and maintain healthy habits in their children with ND/MHD, in order to disseminate these strategies to other parents, clinicians, and other consulting professionals. Five major categories of health behavior parenting strategies emerged during interviews, including 1) allowing their child or teen to choose from constrained options with room for some negotiation and flexibility, 2) clear, often structural boundaries and consistent routines, 3) positive reinforcement and reward systems, 4) advance planning, preparation and review of scenarios challenging to healthy behaviors, and 5) performance of healthy activities as a family and role modeling of healthy choices by parents/ siblings.

A particularly surprising finding was that parents commonly use animals – either pets or therapy animals – to help maintain healthy habits in their children. Parents reported that walking pets improved children’s physical activity levels, and that allowing children to spend time with animals was used as positive reinforcement for healthy choices. Engagement with animals was therapeutic for child and parent stress relief, and even when families did not have their own pet, parents found creative ways to expose their child to animals (e.g. neighbors’ pets or volunteering at animal shelters). Other studies have found benefits to socialization and other psychosocial outcomes associated with animal-assisted therapy (AAT) [17], however, this is the first to identify a potential role for AAT in improving healthy habits and decreasing chronic disease risk among children with ND/MHD.

Given the chronic disease disparities documented in these children and the barriers to shaping healthy habits experienced by their parents, it is critical to increase access to the types of facilitators identified by parents in this study, and disseminate and better support the successful strategies they are employing. Increasing access to multi-setting, targeted programming that uses realistic strategies to instill and maintain healthy eating, physical activity and stress management habits is particularly important. If paired with opportunities for parent training, self-care, and/or childcare relief, these types of programming could restore parental reserve capacity and build social capital, rather than further draw upon them.

In order to realistically increase such offerings and meet the needs of children with ND/MHD and their families, however, there is a need for more specialized, adaptive training for health professionals such as registered dietitians, physical education teachers, community exercise specialists, and mindfulness instructors. Without training and the resultant ability to deliver realistic, population-specific guidance, even existing specialist resources will be underutilized by parents. Additionally, future research should utilize comprehensive theoretical frameworks to develop and test more realistic, family-based interventions to improve health habits among these children. In the meantime, clinicians should consider incorporating health parenting education directly into existing parenting courses commonly offered through therapeutic schools and clinics and aimed at parents of children with ND/MHD.

This study is not without important limitations that future research should address. First, selection bias is an amplified concern among parents with diminished reserve capacity and hinders external generalizability. Given that our participants were highly educated and lacked racial/ethnic diversity, our sample is unlikely to capture the full spectrum of parenting challenges faced by this population. Also, while the choice to sample from a therapeutic day school ensured that we reached parents of children with multiple ND/MHD and who engaged in daily parenting of those children, it limited the size of our sampling frame. Thus, that frame could have been exhausted prior to data saturation.

## Conclusions

Our results spotlight how challenging parenting healthy habits in children with ND/MHD can be, given competing demands on parenting resources such as ensuring school attendance, treatment adherence, and safety. Parents perceive existing community health resources and programming to lack practical applicability given their children’s challenges. Therefore, in order to reduce chronic disease disparities and promote health in this population, future research must better adapt existing health promotion materials and programs to more practically support parents in multiple settings including home, schools and community organizations. Innovative approaches are needed to target health behavior change in children with ND/MHD without additional burden on parents, or which help to build social capital and alleviate existing demands on parenting resources.

## Additional file

Additional file 1: Interview Guide. (DOCX 19 kb)

## Data Availability

Due to the small sample size and potential for privacy violations despite de-identification, the data set for this study (interview transcripts) is not available to the public. However, the interview guide is provided as supplementary materials.
