# Supplementary material for: Shaping healthy habits in children with neurodevelopmental and mental health disorders: parent perceptions of barriers, facilitators and promising strategies
Source: Int J Behav Nutr Phys Act. 2019 Jun 26;16:52. doi: 10.1186/s12966-019-0813-6 (PMC6595579; doi:10.1186/s12966-019-0813-6)
Supplement: Supplementary file 1 — Interview Guide. (DOCX 19 kb) [file 12966_2019_813_MOESM1_ESM.docx]

**Supplemental Materials:**

**Interview Guide**

1. Can you tell me a little bit about your family?
   1. How many children do you have?
   2. How many are living with you now?
   3. What is their age range?
   4. How many attend [SCHOOL]?  In what grade(s)?
   5. Do you have any other children with special needs?
2. At present, is there is anything in your home environment that would be important to know about to better understand your interactions with your child about their health behaviors?
   1. E.g., Recently started at [SCHOOL]?  A sibling left home?
3. If a home/family health program was developed at school, what would you like that to look like?
   1. What kinds of support would be helpful to you as a parent?
   2. What kinds of healthy behaviors would you like it to target?
   3. What set up or format would be important for your family (e.g., online, in person, implemented only during the school day)?

Explain that you are going to switch gears a little and talk about your child’s health behaviors in general.  By health behaviors you mean – diet, physical activity, sleep and media use.

1. Which two behaviors are you most concerned about in your child?
   1. Healthy eating
   2. Getting enough exercise
   3. Getting a good night’s sleep
   4. Screen time and media use

Note: Focus on one behavior at a time.  Ask the parent select the behavior they would like to start with.

1. Can you explain why you chose that behavior?
2. What challenges does your child face with respect to this health behavior?
3. How does your family respond to these expressions of challenges?
4. Do you think your child’s (state selected behavior, i.e., diet, PA, sleep, media behavior) is similar to or different from typically developing children of the same age and gender?
5. Has your child’s (behavior) changed in the target child over time (as they have gotten older)?
6. Is this behavior similar to or different from other children in their family?
7. When you are trying to guide or shape your child’s (behavior), what is hardest for you?
8. What strategies do you use to encourage healthy (name behavior) in your child?
9. Which strategies are most effective?
10. Were the strategies implemented as a family or just for this child?
11. Did the effectiveness of strategies change over time or in different contexts?
12. What makes it difficult for you to use these strategies?
13. Is there another parent (biological or non-biological) living in the home with you?
14. If yes, can you describe what role your child’s other parent takes in parenting these types of health behaviors?
    1. Do the two of you have different ideas or beliefs about how to help your child (thinking about this behavior)?
    2. Do you use different parenting strategies to promote the behavior?
    3. Does trying to manage this particular behavior in your child create stress or conflict in your family?
       1. If yes, please describe. How do you resolve these conflicts?

The next questions ask about your family’s quality of life.

1. How would you rate the overall level of stress in your family?
2. Are there any people you rely on to help you care for your child?
3. Do you share responsibility with other family members (e.g., grandparents)
   1. If yes, what does this look like?
4. Are there things you do to support quality of life (and manage stress) in your family? For example, think of things like meditation, yoga, mindfulness, time in nature etc, regular time away.
   1. If so, who is involved (family, only mom, both parents)?
   2. How do you schedule these activities in your day or week?
   3. To what extent have these practices/strategies been helpful or detrimental?
   4. How do your children respond?
5. We are thinking about adding a home component to the [SCHOOL] Moves program that focuses on mindfulness. Does anyone in your family practice mindfulness?
   1. Is this something your family might be interested in?  If yes, do you think your family could successfully use mindful practices at home on a daily basis?
   2. What barriers do you see to mindful practices in your home?
